# ^68^Ga-PSMA-11 PET/CT Initial Staging in Black and White South African Males with ISUP Grade Group 1 and 2 Prostate Adenocarcinoma

**DOI:** 10.3390/biomedicines10040882

**Published:** 2022-04-12

**Authors:** Letjie C. Maserumule, Kgomotso M. G. Mokoala, Christophe van de Wiele, Gbenga Popoola, Khanyisile N. Hlongwa, Honest Ndlovu, Alex Maes, Mariza Vorster, Mike M. Sathekge

**Affiliations:** 1 Department of Nuclear Medicine, Steve Biko Academic Hospital, University of Pretoria, Pretoria 0001, South Africa; letjie.maserumule@gmail.com (L.C.M.); kgomotso.mokoala@up.ac.za (K.M.G.M.); cvdwiele@hotmail.com (C.v.d.W.); khanyi29@gmail.com (K.N.H.); ndlovuhonest@gmail.com (H.N.); alex.maes@azgroeninge.be (A.M.); marizavorster@gmail.com (M.V.); 2 Department of Diagnostic Sciences, University Ghent, 9000 Ghent, Belgium; 3 The Wolds, Discovery House, Lincolnshire NHS Partnership Foundation Trust, Lincoln LN1 1FS, UK; g.popoola45@gmail.com; 4 Department of Morphology and Medical Imaging, Katholieke Universiteit Leuven, 8500 Leuven, Belgium; 5 Nuclear Medicine Research Infrastructure (NuMeRI), Steve Biko Academic Hospital, Pretoria 0001, South Africa

**Keywords:** prostate cancer, ^68^Ga-PSMA PET/CT, PSMA, racial differences

## Abstract

Prostate adenocarcinoma (PCa) is a leading cause of mortality. Black males with high-risk PCa have a poorer prognosis compared to white males. Patients with International Society of Urological Pathology (ISUP) Grade Group (GG) 1 and 2 PCa have little potential for metastases post radical prostatectomy. ^68^Gallium prostate specific membrane antigen (^68^Ga-PSMA) PET/CT imaging for metastatic PCa is superior to conventional imaging in staging high-risk PCa. No strong evidence is available to support imaging low-risk patients. We aimed to evaluate the value of ^68^Ga-PSMA PET/CT in black and white South African (BSA and WSA) males with GG1 and 2 PCa at initial staging. We evaluated 25 WSA and 123 BSA males. The image findings were correlated with prostate specific antigen (PSA). PSA levels significantly correlated with both primary tumor and whole-body PSMA-tumor volume (PSMA-TV) and were higher in BSA males. No differences were noted in the occurrence of metastases; however, PSA, seminal vesicle invasion and black race predicted metastases. Our findings suggest higher PSMA expression and tumor burden in BSA with histologically low-risk PCa, and future research with immunohistochemistry evaluation will be essential to confirm these findings.

## 1. Introduction

Prostate adenocarcinoma (PCa) is the commonest non-cutaneous cancer in males [1]. Worldwide incidence is 29.3 per 100,000, whereas Southern Africa has an incidence of 64.1 per 100,000 [2]. Early identification of PCa has become possible with widespread screening using a serum prostate specific antigen (PSA). Morbidity and mortality from PCa vary per population and the highest mortality is seen in black males of Sub-Saharan Africa and the Caribbean [1,2].

Prostate specific membrane antigen (PSMA) is a type II transmembrane glycoprotein overexpressed in most prostate cancer cells [3]. Positron emission tomography with computed tomography (PET/CT) using ^68^Ga-PSMA ligands internalized in PCa cells takes advantage of the PSMA overexpression. ^68^Ga-PSMA-11 is currently the most widely used molecule [3]. Multiple studies have compared PSMA PET/CT sensitivity to multiparametric magnetic resonance imaging (mpMRI) and histopathology. Basha et al. demonstrated ^68^Ga-PSMA-11 PET/CT sensitivity of 96% with a change in stage seen in 29% [4]. Berger et al. showed the superiority of PSMA PET/CT to mpMRI with 100% detection in the prostate gland and better sensitivity in pelvic nodes’ detection of 81% versus 64% [5]. A meta-analysis demonstrated the PSMA-PET/MR pooled sensitivity of 95% in the detection of the primary tumor [6]. These findings make PSMA imaging a favorable modality in primary prostate and pelvic nodal metastases imaging.

A recent multicenter trial demonstrated superior accuracy and sensitivity of ^68^Ga-PSMA PET/CT over conventional CT and bone scintigraphy in staging high-risk prostate cancer patients [7]. The current imaging guidelines recommend bone scintigraphy and CT for initial staging of high-risk prostate cancer with no strong evidence for the benefit in imaging patients with low- and intermediate-risk disease [8].

The relevance of ^68^Ga-PSMA PET/CT is not well established in low-risk prostate cancer patients [9]. In view of the known aggressive nature of prostate cancer in black males, metastases may occur early even in low-risk groups. Studies have shown very good prognosis with the rare occurrence of metastases post radical prostatectomy in patients with International Society of Urological Pathology (ISUP) grade groups (GG) 1 and 2 PCa demonstrating no biochemical recurrence after 5 years in 96 and 88%, respectively [10,11,12]. The higher grades demonstrate 5-year progression-free survival of between 63% and 23% [10]. The proposal from these data is that GG 1 and 2 patients be integrated into low risk, 3 and 4 into intermediate and 5 into high risk [12]. The aim of this retrospective study was to evaluate the value of early ^68^Ga-PSMA PET/CT in black South African (BSA) and white South African (WSA) men with GG 1 (Gleason 3 + 3) and 2 (Gleason 3 + 4) PCa.

## 2. Materials and Methods

### 2.1. Patient Population

A total of 155 ^68^Ga-PSMA-11 PET/CT scans of GG 1 and 2 treatment-naïve PCa patients were performed at Steve Biko Academic Hospital (Pretoria, South Africa) between January 2016 and January 2021. Inclusion criteria were biopsy-proven tumor in treatment-naïve patients who had a PSA within four weeks of the PET/CT. Seven patients were excluded due to incomplete data.

### 2.2. ^68^Ga-PSMA PET/CT

^68^Ga-PSMA-11 was prepared in house as previously described by our group [13]. An average of 1.85 MBq per kilogram ^68^Ga-PSMA-11 was injected intravenously. The median injected activity was 133 MBq (range, 59.2–247.9 MBq). Radiochemical purity of injected radiopharmaceutical was above 95% in all participants. Whole-body PET/CT imaging from vertex to mid-thigh was performed at 60 min post-injection (range, 60–70 min). A low-dose non-diagnostic CT without contrast was performed for anatomical localization and attenuation correction. There was no special preparation prior to imaging. Patients were requested to void fully just prior to imaging beginning. All patients were imaged on a Biograph 40 Truepoint PET/CT scanner (Siemens Medical Solution, Malvern, PA, USA). Image reconstruction was completed with an ordered subset expectation maximization iterative reconstruction algorithm (four iterations, eight subsets). A Gaussian filter was applied at 5.0 mm FWHM.

### 2.3. Image Analysis

The images were reviewed by two experienced nuclear physicians. The image analysis was completed using a dedicated workstation equipped with Syngo software (Siemens Medical Solutions, Malvern, PA, USA). Qualitative and semi-quantitative analyses of ^68^Ga-PSMA PET/CT findings were performed. Lesions with uptake greater than background were considered PSMA-positive, except for structures with known non-malignant PSMA uptake such as lacrimal glands, salivary glands, liver, spleen gastrointestinal tract and sympathetic ganglia. PSMA-positive findings in the prostate, lymph nodes, soft tissue viscera and skeleton were documented. For all primary prostate gland lesions, the mean and maximum standard uptake values (SUVmean, SUVmax) were determined. The PSMA tumor volumes (PSMA-TV) of the primary and metastatic lesion were determined in volumes of interest with the adapted isocontour threshold of 41%. Total lesion PSMA (TL-PSMA) was calculated by multiplying SUVmean and PSMA-TV. Per patient summation of all lesions PSMA-TV was completed to obtain whole-body PSMA-TV. The size of the lesions was measured on the corresponding low-dose CT. The semiquantitative data were compared between white and black males.

### 2.4. Statistical Analysis

A statistical analysis was performed using the commercially available software package SPSS 28.0 (IBM Corp, Armonk, NY, USA). Normalcy was assessed by means of the Kolmogorov–Smirnov test. For non-normal distributed data, the Mann–Whitney test and Spearman-rank test were used when appropriate. For normally distributed data, Student’s t-tests and the Pearson correlation test were used when appropriate.

Binomial logistic regression was performed to assess variables that predict the presence of malignant involvement outside the prostate (dependent variable) on the ^68^Ga-PSMA PET/CT examination. *p*-values ≤ 0.05 were considered significant.

## 3. Results

### 3.1. Clinicopathological Data

Patient characteristics are shown in Table 1. One hundred and forty-eight patients were included in the study. The mean age was 66.0 years (SD:8.3 years). There were 123 black men and 25 white men. Seventy-eight patients presented with GG 1 and seventy patients with GG 2 prostate cancer. Twenty-nine patients presented with seminal vesicle involvement, thirty-one with lymph node (LN) involvement, fifteen with skeletal metastases and five patients with visceral metastases. The visceral metastases involved the liver in three, lungs in two and brain in one patient.

### 3.2. ^68^Ga-PSMA PET/CT Analysis

There were no differences between patients presenting with GG 1 versus those presenting with GG 2 with regards to the frequency of the presence of seminal vesicle involvement (13/78 versus 16/70, *p* = 0.343), of LN involvement, (14/78 versus 53/70, *p* = 0.344), of skeletal metastases (9/78 versus 6/70, *p* = 0.550) and of visceral metastases (2/78 versus 3/70, *p* = 0.563).

**Figure 1:**
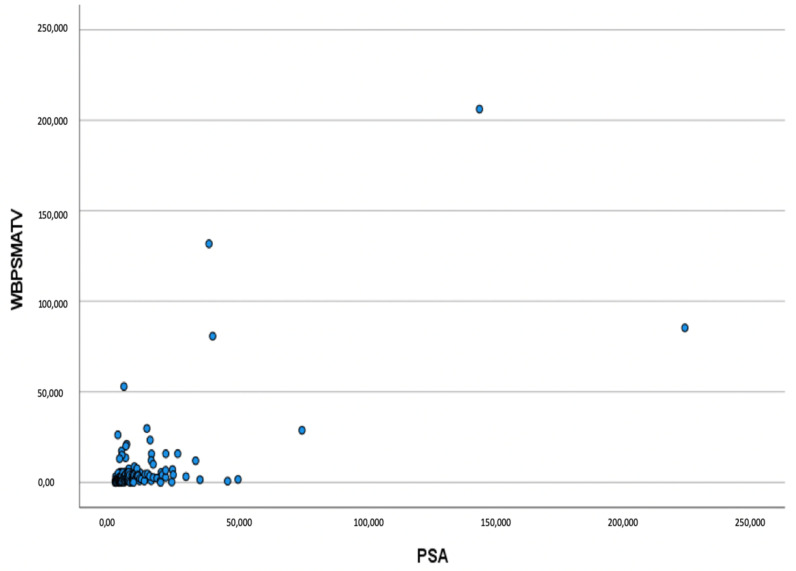
Scatterplot showing the correlation between baseline PSA (ug/L) levels and whole-body PSMA-TV (WBPSMATV).

Baseline PSA levels proved significantly correlated to both the whole-body PSMA-TV (r = 0.676, *p* = 0.0001) and primary tumor PSMA-TV (r =0.624, *p* = 0.0001). See Figure 1.

### 3.3. Comparison of BSA and WSA Males

The baseline PSA value was significantly higher in BSA, with a median value of 50.00 in BSA (IQR 21.37–119.44) versus 29.06 (IQR 7.86–49.23) (*p* = 0.003).

#### 3.3.1. Primary Tumor

**Figure 2:**
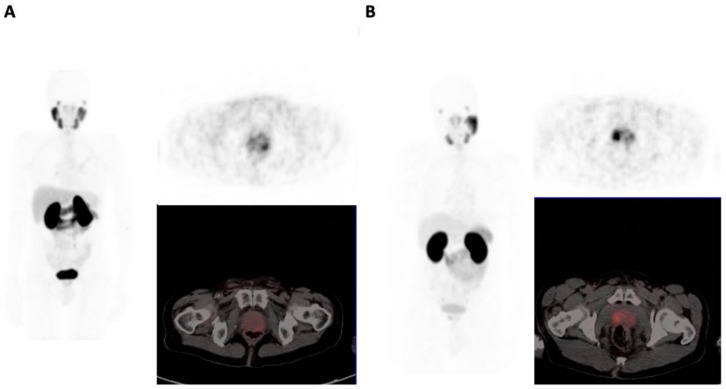
Comparison of primary tumor PSMA-TV in WSA (**A**) and BSA (**B**) for two patients with GG 1 disease in maximum projection image, PET only and fused PET/CT. (**A**) corresponds to a 61-year-old WSA with PSA 73.31 ug/L. SUVmax–3.42, TL-PSMA–0.90, PSMA-TV of 0.29. (**B**) corresponds to a 60-year-old BSA with PSA of 22 ug/L. SUVmax–6.85, TL-PSMA–17.3, PSMA-TV-4.89.

The prostate gland SUVmax and SUVmean (mean (SD)) proved significantly different between BSA and WSA: 15.6 (12.7) versus 8.0 (6.2) (*p* = 0.004) and 5.7 (3.9) versus 3.6 (1.3) (*p* = 0.009), respectively (Figure 2). Furthermore, the median prostate TL-PSMA was significantly higher in BSA 109.43 (IQR 363.12–269.21) versus 23.80 (IQR 5.82–62.48) (*p* < 0.001). There were no significant differences in the frequency of GG 1 and GG 2 (9/26 versus 61/123, *p* = 0.215), and the presence of seminal vesicle invasion (2/25 versus 27/123, *p* = 0.109) between the two groups.

#### 3.3.2. Metastatic Involvement

**Figure 3:**
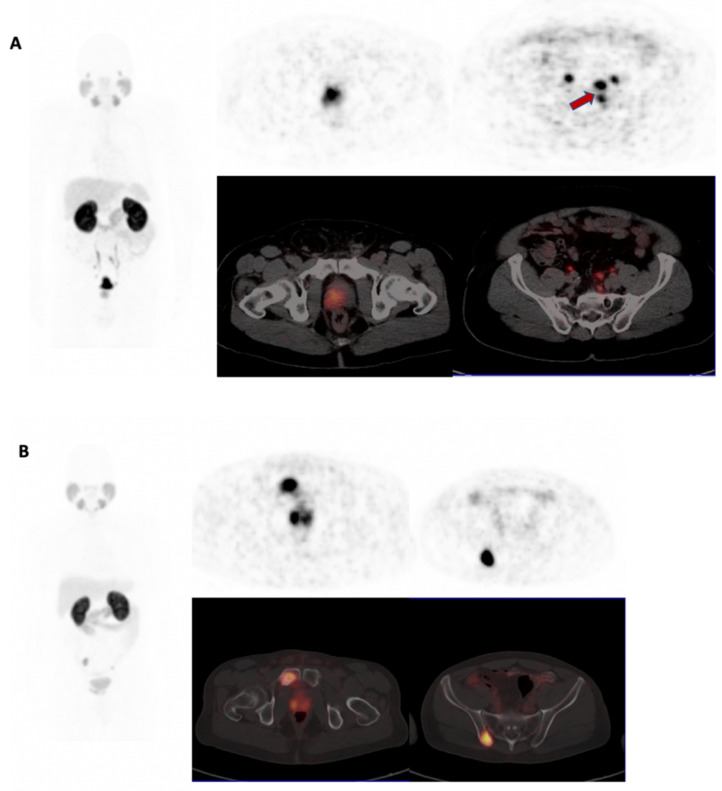
Comparison of whole-body (WB) PSMA-TV in WSA (**A**) and BSA (**B**) for two patients with GG 2 disease. Left: maximum intensity projection image; middle column: PET and fused PET/CT of prostate; right column: PET and fused PET/CT metastases. (**A**) 58 year-old WSA with PSA 59 ug/L, with pelvic nodal metastases (red arrow). Bilateral ureteric tracer excretion seen, WBPSMA-TV-45. (**B**) 57-year-old BSA with PSA of 30 ug/L demonstrating pelvic skeletal metastases, WBPSMA-TV–58.

The presence of LN involvement (6/19 versus 25/98, *p* = 0.681), skeletal metastases (3/25 versus 12/123, *p* = 0.735) and visceral metastases (1/25 versus 4/119, *p* = 0.850) were not statistically different between the two groups. However, the median whole-body PSMA-TV was significantly higher (*p* = 0.004) in BSA 28.24 (IQR 12.03–51.83) versus 10.87 (IQR 2.18–26.87) (see Figure 3).

#### 3.3.3. Factors That Predicated for Extra-Prostatic Involvement

Significant variables predictive for the presence of lymph nodes and/or distant metastases on ^68^Ga-PSMA PET/CT were the presence of seminal vesicle involvement, PSA level at the time of imaging and race (Table 2). For each unit increase in PSA, the probability of LN and/or distant metastases increased by 0.7%. WSA with PCa GG 1 or GG 2 were three-times less likely, and patients without seminal vesicle involvement six-times less likely, to present with LN and/or distant metastases at the time of diagnosis.

## 4. Discussion

Our study aimed to investigate the difference between BSA and WSA with GG 1 and 2 PCa on ^68^Ga-PSMA PET/CT. We showed significantly higher PSA, primary tumor SUVmax, SUVmean, PSMA-TV and TL-PSMA in BSA. Furthermore, BSA had a three-times higher risk of PCa metastases. Other factors that predicted for metastases were seminal vesicle involvement and PSA level. The GG did not predict for the presence of metastases in either group. The present results demonstrate higher ^68^Ga-PSMA-11 uptake and PSA values in BSA with histologically low-risk PCa.

To the best of our knowledge, no previous study investigating the utility of ^68^Ga-PSMA PET/CT in low-risk PCa at initial staging has been performed. Our group previously investigated the role of ^68^Ga-PSMA PET/CT imaging in the initial staging of all PCa grade groups comparing BSA to WSA males [14]. The study showed a correlation of the GG with prostate gland PSMA expression. Furthermore, similar to the present study, the primary tumor SUVmax and serum PSA were significantly higher in BSA, and PSA values correlated with primary tumor PSMA expression. Other studies have also demonstrated a correlation between PSA and PSMA expression [15,16]. The similarities in these findings may imply that even in lower risk PCa, BSA have higher PSMA expression. PSMA expression has been previously shown to be an independent prognostic marker at initial staging, with patients displaying higher expression presenting with higher rates of disease recurrence post radical prostatectomy [17]. The whole-body PSMA-TV proved higher in BSA patients. This finding was due to the presence of a greater local disease extent in these patients, suggesting ISUP CG1 and 2 prostate carcinoma lesions in black patients may have a different, more aggressive biological behavior. The clinical implications of these results for patient management are not yet clear; however, it may be important in risk stratification at initial staging and approach to personalized patient management.

The finding that seminal vesicle involvement is predictive for metastases agrees with the already published literature. It has been shown that seminal vesicle involvement confers poorer prognosis compared to prostate-confined disease, with higher chances of biochemical recurrence after radical prostatectomy [18]. These findings were on magnetic resonance imaging (MRI) and to the best of our knowledge the finding has not been shown on ^68^Ga-PSMA PET/CT imaging. Although PET/CT has limited spatial resolution compared to MRI, the use of ^68^Ga-PSMA PET/CT as a functional biomarker prior to surgery has the advantage of whole-body imaging and better sensitivity to size insignificant early lymph node metastases over MRI. This makes the modality attractive in initial staging to plan curative therapy and avoid early biochemical recurrence.

Multiple studies have proposed different reasons for the difference in the natural history of prostate cancer in black and white males [19,20,21,22,23]. Krimphove et al. found that black males had worse overall survival when they presented with advanced prostate cancer; however, they fared better when they accounted for differences in access to care, treatment and cancer characteristics [19]. Similarly, Riviere et al. conducted a longitudinal study of men with PCa treated through the Veterans Affairs health system, which is an equal-access medical system [23]. They found that African American men did not present with more advanced disease or experience worse outcomes compared to non-Hispanic white men. The findings suggest the late presentation and worse outcomes in black males are related to poor access to healthcare and treatment differences; however, aggressive tumor biology in African men has also been suggested as a contributing factor [24]. South Africa (SA) has one of the highest socio-economic disparities globally and access to health care among the poor is still limited. The country does not have a mass screening program, but advocates for selective screening in high-risk patients; however, Spencer showed that screening by non-urologist doctors is very low in SA [25]. Pitiable acceptance of screening in poorer communities with lower levels of education has also been proven [26]. Black South Africans are still the majority of the patients that come from previously disadvantaged backgrounds with poorer education levels and access to healthcare. This likely explains why, in the present study, they presented with more advanced disease. Thus, public health efforts to improve screening for early detection are necessary. In addition, further investigation into biological factors contributing to the disparities is desirable.

The current imaging guidelines recommend the use of bone scintigraphy and CT for the initial staging of high-risk prostate cancer with no strong evidence for the benefit in imaging patients with low- and intermediate-risk disease [8]. In view of the higher risk of metastatic prostate cancer with black males, the addition of ^68^Ga-PSMA PET/CT may change management in lower GG patients. Further research in this regard is necessary. Widespread acceptance of conservative management strategies in selected patients with GG 1 and GG 2 PCa has been adopted in many centers [27,28,29]. Two approaches are used in early localized prostate cancer diagnosis, namely watchful waiting and active surveillance. Watchful waiting involves giving non-curative androgen deprivation therapy when symptomatic progression occurs, and active surveillance is the provision of curative therapy at signs of progression [30]. Both strategies require frequent rectal examinations, PSA measurements and morphological imaging modalities to detect early progression. In view of the high burden of the public health sector in SA and more advanced disease at diagnosis in BSA, these approaches are unlikely to be feasible. In areas of access to ^68^Ga-PSMA PET/CT, these patients may benefit from early imaging at initial staging.

This study is limited by the small sample size and more-so by the number of white South Africans included in the study, thereby increasing the risk of a type II statistical error when comparing both populations. The small number of white South Africans included is; however, in line with the racial demographics of South Africa, in which this number is smaller than the number of black South Africans.

## 5. Conclusions

Higher SUVmean and max ^68^Ga-PSMA-11 values of primary prostate adenocarcinoma were found in BSA patients, suggesting a higher primary PSMA-lesion density in these patients requiring future histological proof. Whole-body PSMA-TV proved higher in BSA patients due to the presence of a greater local disease extent in these patients, suggesting ISUP GG1 and 2 prostate adenocarcinoma lesions in black patients may have a different, more aggressive biological behavior. The clinical relevance of this finding in terms of BSA prostate adenocarcinoma patients, their initial staging and treatment management warrants further evaluation.

## Figures and Tables

**Table 1:** Patient characteristics.

	Study Group			
	White Males (25)		Black Males (123)	
	Patient Characteristics	Prostate-Confined on PET/CT *n* (%)	Patient Characteristics	Prostate-Confined on PET/CT *n* (%)
Age (years)	66.6		65.85	
mean (range)	(47–78)		(47–87)	
Pre-scan PSA				
<10 *n* (%)	7 (28)	6 (86)	9 (7)	9 (100)
10–20	3 (12)	3 (100)	16 (13)	13 (81)
>20	15 (60)	9 (60)	98 (80)	71 (72)
ISUP grade group				
1 *n* (%)	16 (64)	4 (25)	63 (51)	50 (41)
2 *n* (%)	9 (36)	3 (33)	60 (49)	44 (36)

ISUP: International Society of Urological Pathology; *n*: number of patients; PET/CT: positron emission tomography/computed tomography; PSA: prostate specific antigen; %: percentage.

**Table 2:** Logistic regression of variables that predict for metastases.

	Exp(B) (95% C.I)	*p* Value
GG	0.874 (0.359–2.127)	0.767
Age	0.989 (0.938–1.043)	0.689
PSA	1.007 (1.002–1.011))	0.004
Race	0.320 (0.103–0.998)	0.050
Seminal vesicles	0.146 (0.052–0.411)	0.000

GG: grade group; PSA: prostate specific antigen.

## Data Availability

The data presented in this study are available upon request from the corresponding author. They are not publicly available due to patient confidentiality.
